# Muscle fatigue affects dynamic tibiofemoral movements during jumping tasks in healthy participants

**DOI:** 10.1002/jeo2.70794

**Published:** 2026-05-29

**Authors:** Senja Prince, Reinoud W. Brouwer, Tom Vendrig, Vivian A. den Hertog, Michèle N. J. Keizer

**Affiliations:** ^1^ Department of Human Movement Sciences University Medical Center Groningen, University of Groningen Groningen The Netherlands; ^2^ Department of Orthopedic Surgery Martini Hospital Groningen Groningen The Netherlands

**Keywords:** anterior cruciate ligament injury, anterior tibia translation, internal tibia rotation, kinematics, knee biomechanics

## Abstract

**Purpose:**

The main function of the anterior cruciate ligament (ACL) is to restrain anterior tibia translation (ATT) and internal tibia rotation (ITR) relative to the femur. Muscle fatigue has been identified as a potential risk factor for ACL injury due to its negative effects on neuromuscular control, proprioception, and joint stability. The aim of this study was to determine the influence of muscle fatigue on dynamic ATT (ATTd) and dynamic ITR (ITRd) during the single‐leg hop for distance (SLHD) and side hop (SH) in healthy participants.

**Methods:**

Twenty healthy participants were included. ATTd, ITRd and knee flexion angle were assessed during the SLHD and SH using a 3D motion capture system in a nonfatigued and fatigued condition. Fatigue was induced by 30 s of squat jumps before each task. ATTd, ITRd and knee flexion angle were analysed using statistical parametric mapping (SPM) to compare conditions, from initial contact (IC) to 0.75 s after IC.

**Results:**

During the fatigued condition, a significant increase in ATTd was observed for both the SLHD (*p* = 0.002; 0.06–0.38 s after IC) and the SH (*p* = 0.02; 0.15–0.36 s after IC). No significant differences were observed in ITRd during both tasks. A significant increase in knee flexion angle was observed during the fatigued condition for the SH (*p* = 0.048; 0.11–0.15 s after IC), but not for the SLHD.

**Conclusion:**

Muscle fatigue resulted in increased ATTd, offering new insights into intra‐articular knee kinematics in fatigued conditions. The observed changes were not multiplanar and did not match timing typically associated with ACL injury mechanisms, leaving the relevance to injury risk unclear.

**Level of Evidence:**

Level II.

**Abbreviations**
ACL: anterior cruciate ligament
ANOVA: analysis of variance
ATT: anterior tibia translation
ATTd: dynamic anterior tibia translation
CTc: Central Ethics Committee
IC: initial contact
ITR: internal tibia rotation
ITRd: dynamic internal tibia rotation
SH: side hop
SLHD: single‐leg hop for distance
SnPM: statistical nonparametric mapping
SPM: statistical parametric mapping

## INTRODUCTION

Despite increasing research on anterior cruciate ligament (ACL) injury prevention [7], the incidence of ACL injuries continues to rise. In 2016 the rate was estimated at 68.6 per 100.000 persons per year in the United States [53], from which approximately two‐thirds resulted from noncontact mechanisms [11]. If implementation of injury prevention protocols could reduce the incidence of ACL injuries and consequently the need for ACL reconstructions by 50%, this could save more than $1.1 billion in medical costs in the United States annually [26]. In addition, ACL injuries have a great effect on the long‐term quality of life due to the low return‐to‐sport rate and increased risk of early‐onset osteoarthritis [2, 41]. Therefore, it is crucial to identify high‐risk factors that alter biomechanics and increase the risk of noncontact ACL injuries, for developing effective injury prevention protocols.

Muscle fatigue has been proposed as a potential risk factor for ACL injuries [1]. However, epidemiological studies have reported inconsistent injury‐timing patterns during games [16, 22, 24], leaving the role of fatigue in injury occurrence unclear. In contrast, experimental and biomechanical studies more consistently report fatigue‐related alterations in movement patterns that are considered to increase injury risk [1], including changes in knee and hip flexion angles [4, 8, 39]. Several of these kinematic changes suggest inefficient landing techniques [20], which can result in stiffer landings and potentially increased loading of the ACL [36].

However, previous studies have primarily examined fatigue‐related changes in gross hip and knee angles, whereas dynamic tibiofemoral movements—that is, anterior tibial translation (ATT) and internal tibial rotation (ITR)—have been examined to a lesser extent. In particular, the biomechanical behaviour of the intra‐articular knee joint under fatigued conditions remains poorly understood. Given that the primary function of the ACL is to prevent ATT and ITR [14], an inability to control these tibiofemoral movements could result in increased ACL strain [65], which may be relevant to ACL loading mechanisms. To the best of the author's knowledge, only one study has been conducted on the effect of muscle fatigue on dynamic ATT (ATTd) [58]. However, this study used a CA‐4000 electrogoniometer, which restricts full range of motion and does not allow assessment of dynamic ITR (ITRd). Therefore, a more comprehensive and ecologically valid assessment of dynamic tibiofemoral movements during functional tasks under fatigue is needed, which may contribute to a more detailed biomechanical understanding of knee joint behaviour under fatigued conditions.

In this context, the present study aimed to investigate whether muscle fatigue influences dynamic tibiofemoral movements during high‐impact landing tasks. Specifically, ATTd and ITRd were assessed following a fatigue protocol in healthy participants during the single‐leg hop for distance (SLHD) and the side hop (SH), using optical motion capture to quantify knee kinematics. As a secondary outcome measure, knee flexion angle was evaluated. It was hypothesized that muscle fatigue would result in increased ATTd and ITRd and altered knee flexion angle during dynamic, high‐impact movements.

## METHODS

This cross‐sectional within‐subjects study was conducted between September 2022 and April 2023. The study design, procedure, and protocol received approval from the local ethics committee (IRB number: 202200331) of the University Medical Center Groningen. All participants read and signed informed consent prior to participation.

### Participants

Because no data were available on between‐condition differences in ATTd and ITRd at the time of study design, an a priori sample size calculation for repeated measures analysis of variance (ANOVA) was performed using a correlation of *r* = 0.59 between the mean dynamic ATT over time for both legs [30], an alpha level of 0.05, an effect size of 0.3 and a power of 0.8, for one group and two measurements. Based on this calculation, 20 healthy participants were included in this study, who were recruited from the local university. Participants aged 18–45 years were included. Exclusion criteria consisted of participants with a history of severe lower limb injury, surgery, or neurological dysfunction, and participants who were not proficient in the Dutch language.

### Study Parameters

The primary outcome measures were ATTd and ITRd during the landing phase of the single‐leg hop for distance (SLHD) and the side hop (SH). These tasks were chosen as they lead to significant knee loads, are sport‐specific and are incorporated in return‐to‐sports test battery protocols for ACL‐injured patients [21, 62]. The secondary outcome measure was knee flexion angle. Kinematic data were recorded using a 16‐camera 3D motion capture system at a frequency of 200 Hz (VICON VANTAGE; VICON Motion Systems Ltd.). Outcome measures were assessed in both the participant's dominant leg—defined as the leg typically used to shoot a ball—and non‐dominant leg, to examine potential differences in fatigue‐related effects between limbs.

### Procedure

Each participant was measured in a single session of approximately 2 h, which included both a nonfatigued and a fatigued condition. The participants wore tight, short sports pants and sports shoes with as much grip as possible. First, participants completed a questionnaire about their sports participation. Hereafter, 42 reflective markers were applied on the lower extremities using a marker configuration developed for the assessment of dynamic tibiofemoral movements with minimal influence of soft tissue artifacts [30], as described in Supporting Information S1: Appendix A. All markers were placed directly on the skin, except for the markers on the pelvis, which were placed on the sports pants. A static calibration was performed to define rigid body marker clusters for the femur and tibia to reduce soft tissue artifacts, and dynamic calibrations were performed to identify hip joint centres and knee axes of rotation (see Supporting Information S1: Appendix A for an extensive explanation). A prior sensitivity analysis has shown that marker position errors introduce estimated measurement errors of 1.5 mm for ATTd and 0.76° for ITRd [30], while a prior reliability study indicated excellent reliability of this method for measuring ATTd and ITRd during the SLHD and SH for research purposes [63].

Before measurement, participants practiced the SLHD and SH three times with both legs. The participants were instructed to stand on one leg and hop as far as possible in either the forward direction for the SLHD or the sideways direction for the SH, and land on the same leg. A jump was successful if the participant could maintain a stable position after landing for at least three seconds, without shoving the foot. The median distance from the practice jumps was used as the starting distance from the middle of a 40x60cm force platform (AMTI BMS400600HF‐OP‐2K system; Advanced Mechanical Technology Inc.; 2000 Hz) during the testing procedure and was consistent for both conditions.

The testing procedure is shown in Table 1. Participants received the same instructions as during the practice trails, after which they performed three successful repetitions of each task (SLHD and SH) with both legs, defined as the nonfatigued condition. Trials were repeated if participants failed to maintain a stable stance after landing, if the foot did not land fully on the force platform, or if large marker gaps were present. Following the nonfatigued condition, participants performed 30 s of squat jumps to induce muscle fatigue prior to each jumping task (SLHD and SH). Participants were instructed to perform as many squat jumps as possible within 30 s, while jumping as high as possible and bending their knees as much as possible during the downward phase. Three successful repetitions of both tasks (SLHD and SH) started as soon as possible after the squat jumps, which were defined as the fatigued condition. The starting leg and the overall order of the SLHD and SH was randomized for each participant, but remained consistent within participants across the nonfatigued and the fatigued condition.

**Table 1:** Order of the jumping tasks in the protocol.

Practice jumps		Nonfatigued condition		Fatigued condition			
Three repetitions of SLHD	Three repetitions of SH	Three successful repetitions of SLHD	Three successful repetitions of SH	30‐s squat jumps	Three successful repetitions of SLHD	30‐s squat jumps	Three successful repetitions of SH

*Note*: The overall order of the single‐leg hop for distance (SLHD) and side hop (SH) was randomized for each participant. Within participants, this order remained consistent during the nonfatigued and the fatigued condition.

### Data processing

Data were processed and analysed using a customized MATLAB (2022a, The MathWorks Inc.) script. Raw 3D marker position data were filtered with a 10 Hz low‐pass frequency convolution filter and gaps were filled using quadratic spline interpolation with zero lag. ATTd, ITRd and knee flexion angle were computed as described in Supporting Information S1: Appendix A, and were analysed from initial contact (IC) to 0.75 s after IC. IC was defined as the moment when the vertical ground reaction force exceeded 5% of the body weight. This analysed timeframe gives a comprehensive view of the landing phase, corresponding to the period from ground contact until the knee reaches full extension following landing. For each participant and each task, the mean of the successful trials was calculated for both legs separately and used for statistical analysis.

### Statistical analysis

The open‐source spm1d package (v. 0.4, www.spm1d.org, Pataky, 2012) in MATLAB 9.14 (The MathWorks Inc.) was used for statistical analysis of ATTd, ITRd and knee flexion angle. The normality of the data distribution was assessed using the Statistical Parametric Mapping (SPM) Shapiro–Wilk test. If the data followed a normal distribution, an SPM two‐way repeated measures ANOVA was performed to examine potential significant differences between conditions (nonfatigued/fatigued) and potential interaction effects between condition and jumping leg (non‐dominant/dominant). If the data did not follow a normal distribution, a statistical nonparametric mapping (SnPM) two‐way repeated measures ANOVA with permutations was conducted. The within‐subject factors were the condition (nonfatigued/fatigued) and jumping leg (nondominant/dominant), and the dependent variables were ATTd, ITRd and knee flexion angle from IC to 0.75 s after IC of the landing. When the SPM test statistic exceeded the critical *F*‐value based on an alpha of <.05, the null hypothesis was rejected, implying a significant difference between conditions. When a significant cluster was identified, the maximum difference within this region was determined for each participant and each leg and used to compute the mean ± standard deviation (SD) of the maximum difference. To quantify effect sizes, partial *η* ^2^ values were computed. For each timepoint within a significant cluster, the *F*‐statistic from the SPM two‐way ANOVA and the corresponding degrees of freedom were used. Partial *η* ^2^ was computed using the following formula: [35]

$$\eta_{p}^{2}(t)=\frac{F\left(t\right)\;*\;{\mathrm{df}}^{\mathrm{effect}}}{F\left(t\right)\;*\;{\mathrm{df}}^{\mathrm{effect}}+{\mathrm{df}}^{\mathrm{error}}}$$

The resulting values were averaged across all timepoints within the significant cluster, yielding a mean partial *η* ^2^ for each cluster. Effects were interpreted as small (*η* ^2^ < 0.06), medium (*η* ^2^ ≥ 0.06) or large (*η* ^2^ ≥ 0.14) [35]. However, as effect sizes were derived post‐hoc from the SPM F‐statistics, they should be interpreted with caution.

## RESULTS

The characteristics of the participants are presented in Table 2. After data inspection, 9.8% (47/480) of the total number of trails were excluded because of insufficient data quality, due to missing markers. Of the excluded trials, 38% were nonfatigued trials and 62% were fatigued trials.

**Table 2:** Baseline characteristics of participants.

	Men (*n* = 6)	Women (*n* = 14)	Total (*n* = 20)
Age (years)^a^	20.5 ± 2.4	20.3 ± 2.0	20.4 ± 2.1
Weight (kg)^a^	78.6 ± 7.8	64.5 ± 9.4	68.8 ± 11.0
Height (cm)^a^	187.7 ± 5.1	171.6 ± 5.6	176.4 ± 9.3
BMI (kg/m^2^)^a^	22.1 ± 2.7	21.9 ± 3.0	22.0 ± 2.8
Sport activity (hours per week)^a^	8.8 ± 3.8	5.5 ± 2.4	6.5 ± 3.2
Pivoting sports, yes/no (*n*)	3/3	7/7	10/10

^a^ Means ± standard deviation was reported.

For the final analysis, 73% (117/160) of the calculated means were based on three valid trials, 24% (39/160) were based on two valid trials, and 3% (4/160) were based on one valid trial. The mean range of ATTd was 15.6 ± 5.0 mm during the SLHD and 11.0 ± 4.8 mm during the SH. The mean range of ITRd was 8.4 ± 2.6° during the SLHD and 8.3 ± 3.3° during the SH.

### Single leg hop for distance

**Figure 1:**
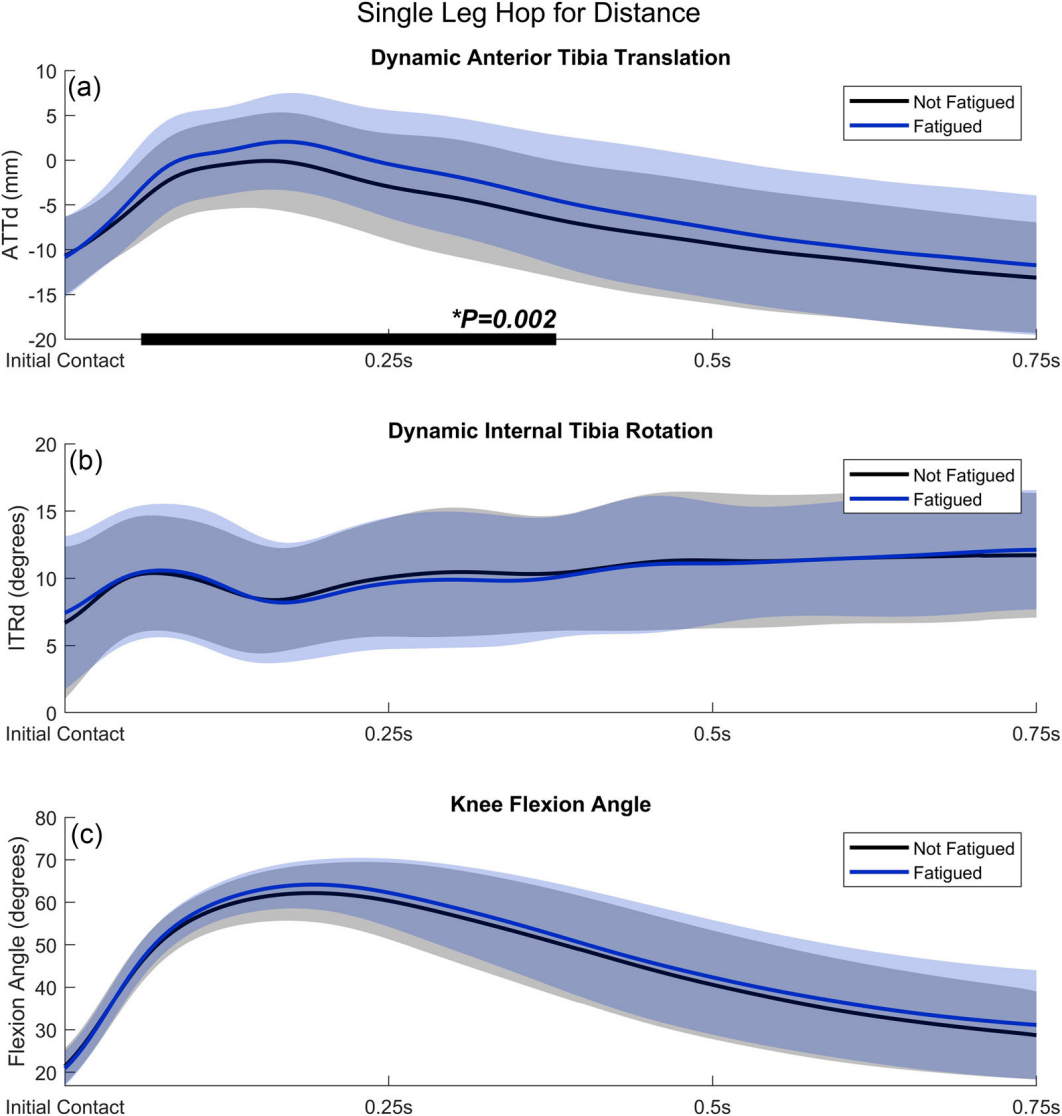
(a) Dynamic anterior tibial translation (ATTd), (b) dynamic internal tibial rotation (ITRd) and (c) knee flexion angle during the single‐leg hop for distance, comparing nonfatigued (black line) and fatigued (blue line) conditions. Light shaded areas represent standard deviations. Instances during which the SPM test statistic exceeded the critical threshold are indicated by black bars along the x‐axis, implying a significant difference between conditions. SPM, statistical parametric mapping.

Figure 1 shows the mean ATTd and ITRd during the SLHD of the nonfatigued and fatigued condition, including significant clusters of the SPM two‐way repeated measures ANOVA analysis. Participants showed significantly more ATTd between 0.06 s and 0.38 s after IC in the fatigued condition compared to the nonfatigued condition (maximal *F*(1, 19) = 18.5; threshold *F* = 8.5; *p* = 0.002; *η* ^2^ = 0.41; Figure 1a). The maximum difference within this timeframe was 4.2 ± 4.1 mm (mean ± SD). In contrast, ITRd was not significantly different between conditions (maximal *F*(1, 19) = 2.8; threshold *F* = 8.2; *p* > 0.05; Figure 1b). Furthermore, knee flexion angle was not significantly different between conditions (maximal *F*(1, 19) = 8.2; threshold *F* 8.5; *p* > 0.05; Figure 1c). For all outcome measures, interaction effects between the condition (nonfatigued/fatigued) and jumping leg (nondominant/dominant) were not significant (*p* > 0.05).

### Side hop

**Figure 2:**
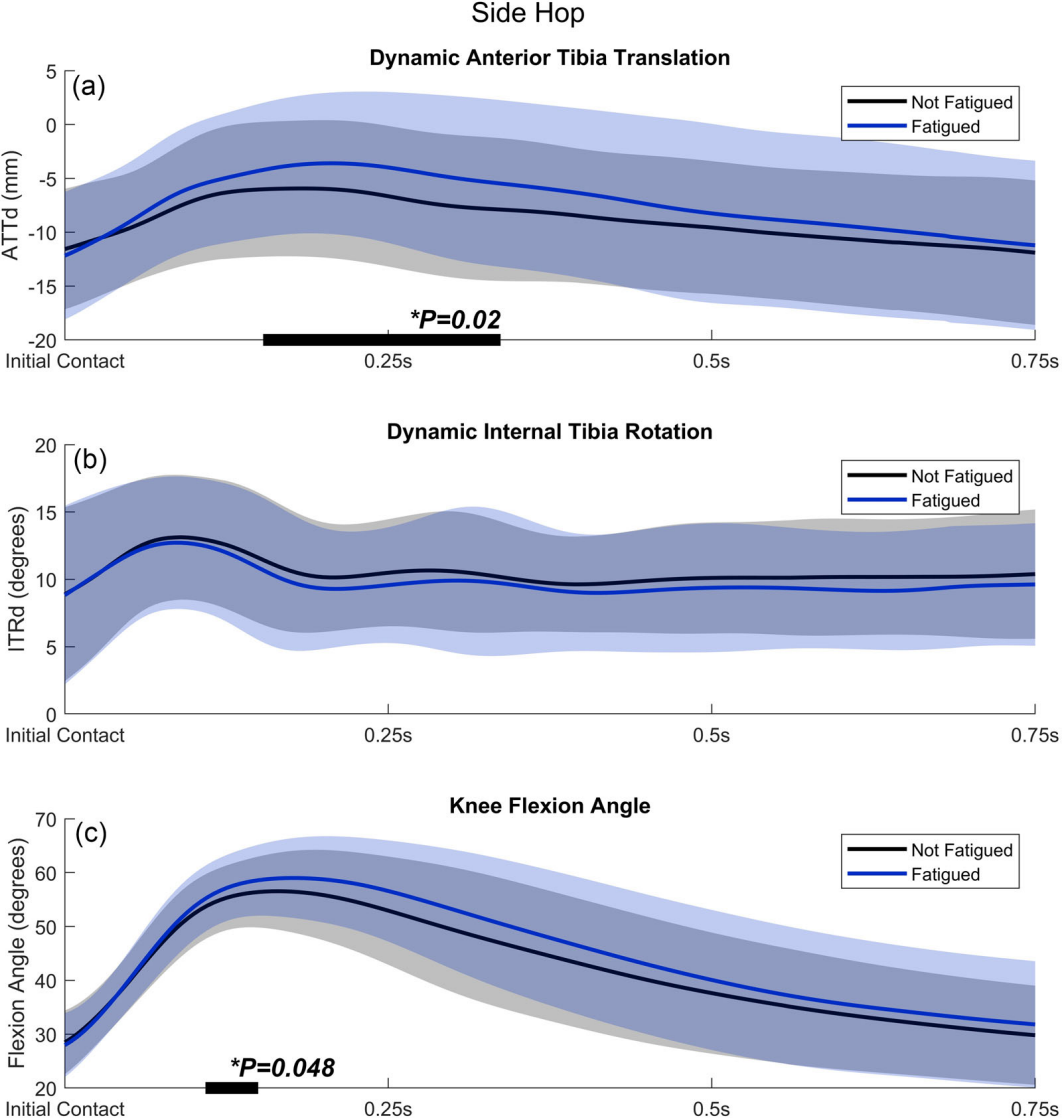
(a) Dynamic anterior tibial translation (ATTd), (b) dynamic internal tibial rotation (ITRd) and (c) knee flexion angle during the side hop, comparing nonfatigued (black line) and fatigued (blue line) conditions. Light shaded areas represent standard deviations. Instances during which the SPM test statistic exceeded the critical threshold are indicated by black bars along the *x*‐axis, implying a significant difference between conditions. SPM, statistical parametric mapping.

Figure 2 shows the mean ATTd and ITRd between the nonfatigued and fatigued condition during the SH, including significant clusters of the SPM two‐way repeated measures ANOVA analysis. Participants showed significantly more ATTd between 0.15 and 0.36 s after IC in the fatigued condition compared to the nonfatigued condition (maximal *F*(1, 19) = 10.8; threshold *F* = 8.3; *p* = 0.02; *η* ^2^ = 0.35; Figure 2A). The maximum difference within this timeframe was 3.8 ± 4.7 mm (mean ± SD). In contrast, ITRd was not significantly different between conditions (maximal *F*(1, 19) = 4.2; threshold *F* = 8.4; *p *> 0.05; Figure 2b). Furthermore, participants showed significantly more knee flexion between 0.11 and 0.15 s after IC in the fatigued condition compared to the nonfatigued condition (maximal *F*(1, 19) = 9.3; threshold *F* = 8.6; *p* = 0.048; *η* ^2^ = 0.32; Figure 2C). The maximum difference within this timeframe was 2.5 ± 3.7° (mean ± SD). For all outcome measures, interaction effects between the condition (nonfatigued/fatigued) and jumping leg (nondominant/dominant) were not significant (*p* > 0.05).

## DISCUSSION AND IMPLICATION

This study investigated whether ATTd and ITRd increase following a fatigue protocol in healthy participants during the SLHD and SH. The findings of this study show that ATTd was significantly greater in the fatigued condition compared to the nonfatigued condition during both tasks, while ITRd showed no significant difference. Furthermore, knee flexion angle was significantly greater in the fatigued condition compared to the nonfatigued condition during the SH.

### Muscle fatigue and knee kinematics

To the best of the author's knowledge, Tagesson et al. [58] is the only prior study examining the influence of muscle fatigue on dynamic ATT in healthy participants, reporting no statistically significant difference in ATTd during gait and one‐legged squats after a fatigue protocol. In contrast to Tagesson et al. [58], the current study found a significant increase of ATTd after muscle fatigue, which could be explained by differences in the performed tasks or methodological differences. During a one‐legged squat, the ground‐reaction force is lower compared to the landing phase of the SLHD and SH, resulting in inferior knee loads. In addition, Tagesson et al. [58] used a CA‐4000 electrogoniometer to measure ATTd, which may have restricted participants movements and potentially altered their landing strategy. The current study used wireless markers, allowing participants to land more naturally, similar to real‐life high‐risk situations outside the laboratory. Since landing technique has a significant influence on lower extremity kinematics and subsequently impacts ACL load [36], this may explain the observed differences in results.

The finding of the current study that fatigue had no impact on ITRd during the SH and SLHD aligns with the results of Hantes et al. [23], who measured ITRd of the weight‐bearing leg during a swinging manoeuvre. However, multiple other studies did find a significant increase of ITRd after muscle fatigue during side‐step cutting tasks [42, 44, 45, 60, 68]. In contrast to the SH and SLHD, the side‐step cutting task includes a change‐of‐direction component, which could have enhanced the effect of muscle fatigue on ITRd. Therefore, the SLHD and SH might not have been sufficiently sensitive to elicit changes in ITRd.

Knee flexion angle did not differ significantly during the SLHD, but showed a significant increase after muscle fatigue during the SH around the timepoint of peak knee flexion angle, consistent with several previous studies [8, 10, 39]. In contrast, other previous studies have reported a significant decrease of knee flexion angle following muscle fatigue [4, 6, 13, 34, 42]. These conflicting results may be explained by differences in task design, as prior studies involved repeated jumping tasks where participants had to perform a subsequent jump immediately after landing [4, 6, 34], or unanticipated side‐cutting task [13, 42]. Furthermore, it should be noted that in the present study, the observed increase in knee flexion angle was brief, lasting only 4 ms.

### Mechanisms underlying the effect of muscle fatigue on tibiofemoral movements

Muscle fatigue can disrupt sensory integration and complex motor planning, which may lead to failure in predicting joint loads [59], and reduce the ability to optimally regulate knee‐joint stiffness and dynamic stability [57]. Previous studies suggest that fatigue‐related changes may be associated with alternations in muscle coordination, such as delayed muscle reflex responses of the hamstrings [5] and reduced hamstrings‐quadriceps co‐contraction [37], which are known to influence dynamic tibiofemoral movements [43]. In particular, greater hamstring force and hamstrings‐quadriceps co‐contraction have been shown to reduce ACL strain by resisting anterior shear forces when the knee is flexed and thereby limiting ATT [43, 61]. Although neuromuscular function was not directly assessed in the present study, these previously described mechanisms provide a plausible physiological explanation for the increased ATTd observed under fatigued conditions in the present study. However future research should include direct measurement of muscle activation patterns, to confirm these hypotheses.

The significant ATTd differences in this study occurred between 0.06 and 0.38 s after IC for SLHD and 0.15–0.36 s after IC for SH. An explanation for the timing of the significant differences observed in this study may be that peak vertical ground reaction force typically occurs shortly before these timepoints [28, 51]. The phase in which the observed differences in ATTd occurred likely reflects the phase when muscle fatigue is most pronounced and neuromuscular control is altered. As a result of muscle fatigue, the knee joint may experience reduced active stabilization and is unable to control the ground reaction force, leading to increased ATTd. Increased anterior‐posterior knee laxity in passive situations has been linked to a higher risk of ACL injury [55], suggesting that greater ATTd may also increase ACL injury risk. However, ACL injuries are known to involve multiplanar loading mechanisms [32], typically combining ATT, ITR and valgus motion. As no significant fatigue‑related changes in ITRd were observed in the present study, the implications of the current findings for ACL injury mechanisms should be interpreted with caution. In addition, it is important to note that this timeframe does not correspond with the typical timing of ACL injuries, which generally occur within the first ~50 ms after IC [3]. Therefore, the direct implications of these findings for ACL injury mechanisms might be limited. However, participants in this study performed controlled movements in a laboratory setting. In real‐life high‐risk situations outside the laboratory, neuromuscular delays, unexpected perturbations, and higher external loads may extend the timeframe over which injurious knee mechanics develop. Furthermore, the observed increase of approximately 4 mm in ATTd appears clinically relevant relative to the mean range of ATTd (15.6 mm during the SLHD and 11.0 during the SH), the large effect sizes, and in relation the commonly used clinical threshold of a side‐to‐side difference of ≥3 mm, which is often considered indicative of abnormal knee laxity [18].

In a prior reliability study using the same measurement method for ATTd and ITRd and a similar study population, participants repeated jumping tasks within 2 h after markers were placed by two different examiners [63]. For ATTd, mean standard error of measurement values were 2.7 and 2.6 mm for the SLHD and SH, respectively. These standard error of measurement values incorporate potential training effects, as the repeated jumps in the reliability study were performed within a similar 2‐h timeframe. In the present study, the observed increase in ATTd after fatigue exceeded these error values. Therefore, the greater ATTd observed in the fatigued condition is unlikely to be explained by measurement errors or training effects, and more likely reflects a true effect of fatigue.

In contrast to our hypotheses, no significant effect of muscle fatigue on ITRd was observed. Besides the tasks in the present study not including a change‐of‐direction component, this finding may also be explained by the use of squat jumps in the fatigue protocol, which likely induced fatigue in the medial and lateral hamstrings to a similar extent. Consequently, fatigue‐related alterations in hamstrings activation may not have produced asymmetrical changes in tibial rotational control. In addition, beyond the stabilizing role of the ACL, it is possible that ITRd relies more strongly on other passive stabilizing knee structures, such as the anterolateral ligament [40], whereas ATTd may rely more on active muscular contributions. As muscle fatigue primarily affects neuromuscular control rather than the mechanical properties of passive structures [52], this may help explain why ITRd was less affected by muscle fatigue than ATTd.

In addition to ATTd, knee flexion angle increased significantly from 0.11 to 0.15 s after IC of the SH, which corresponds to the timeframe slightly before the significant differences in ATTd. Although increased knee flexion is generally considered a safer landing strategy [38], the greater knee flexion angle observed in this study might have elicited increased quadriceps activity as a damping response to limit further knee flexion, thereby increasing ATTd in the subsequent phase of the landing.

### ACL injury screening and prevention protocols

Current ACL injury screening tools largely focus on knee valgus angle, anatomic characteristics, and hamstrings‐to‐quadriceps strength ratio under nonfatigued conditions [46, 48], and show limited predictive ability for ACL injury risk [54]. Similarly, current ACL injury prevention programs target neuromuscular control and strength [27, 47, 50], but do not consistently incorporate fatigued conditions [15, 50]. While the primary focus of the present study is to advance the biomechanical understanding of knee joint behaviour under fatigued conditions, rather than to propose direct changes in clinical ACL injury screening or prevention protocols, the findings may help contextualize potential limitations of current approaches. The present findings of increased ATTd suggest that fatigue may influence movement patterns related to the function of the ACL. Therefore, fatigue‐related alterations in knee kinematics that potentially affect the ACL may not be fully captured by current screening and prevention approaches, which might limit their ecological validity.

### Limitations and future research

Only several studies have specifically addressed ATTd and ITRd in general [9, 29, 30, 31, 58, 62, 66, 67]. A strong point of this study is that it is one of the first to measure tibiofemoral movements dynamically without limiting range of motion and during demanding tasks, using an optical motion capture system. While marker‐based motion capture is widely regarded as the practical standard for 3D kinematic assessment, it is more prone to soft tissue artifacts than fluoroscopy (the gold standard), requiring some caution for the interpretation of ATTd and ITRd. However, employing several techniques to minimize these artifacts, such as using an extensive marker set distributed across the femur and tibia in combination with a rigid body marker configuration [17, 56], and analysing within‐subject differences, enabled dynamic tibiofemoral movements to be measured with sufficient accuracy [30]. It is important to note that this study exclusively investigated ATTd and ITRd, which are closely related to ACL loading [25], but do not directly reflect actual ACL load; therefore, caution is warranted when interpreting their relationship with ACL injury risk.

Additionally, there are some limitations to consider regarding the protocol and study population. First, the study cohort consisted of young, physically active participants with a slightly unbalanced sex distribution, which may affect generalizability. However, this population aligns with the age range in which ACL injury incidence is high [12, 53]. Furthermore, the experimental tasks were pre‐planned and laboratory‑controlled, and the fatigue protocol does not reflect the reactive and cognitively demanding fatigue typically encountered during real‑world sports participation. In addition, incorporating a measure of fatigue—such as the rate of perceived exertion—could help verify whether participants were truly fatigued and account for individual differences in physical fitness. Furthermore, including electromyography would provide a better understanding of the underlying neuromuscular mechanisms affected by muscle fatigue [59].

In previous research, Kim et al. [33] demonstrated that ACL‐injured patients showed a greater decline in knee proprioceptive acuity and quadriceps neuromuscular function under fatigued conditions compared to healthy participants. This suggests that muscle fatigue may have a larger impact on patients with a history of ACL injury. Consequently, further research is needed to investigate how muscle fatigue affects dynamic tibiofemoral movements in patients following ACL reconstruction. If ATTd similarly increases under fatigued conditions in ACL‐reconstructed patients, in combination with other altered lower extremity kinematics [19], it would underscore the importance of incorporating muscle fatigue‐based assessments in return‐to‐sport test batteries, which could enhance the predictive value of return‐to‐sports testing [49, 64].

## CONCLUSION

Muscle fatigue resulted in a significant increase in ATTd during the SLHD and SH, without affecting ITRd, providing novel insight into intra‐articular knee kinematics under fatigued conditions. While these findings suggest that fatigue may influence tibiofemoral control during dynamic tasks, the alterations were not multiplanar, as typically observed in ACL injury mechanisms, and their temporal characteristics do not align with reported injury patterns. Therefore, the relevance of these findings to ACL injury risk remains unclear and warrants further investigation.

## AUTHOR CONTRIBUTIONS

**Tom Vendrig**, **Michèle N. J. Keizer** and **Reinoud W. Brouwer**: Conceptualization. **Senja Prince** and **Vivian A. den Hertog**: Data curation. **Tom Vendrig** and **Michèle N. J. Keizer**: Formal analysis. **Senja Prince** and **Vivian A. den Hertog**: Investigation. **Tom Vendrig** and **Michèle N. J. Keizer**: Methodology. **Michèle N. J. Keizer**: Project administration. **Senja Prince** and **Vivian A. den Hertog**: Resources. **Senja Prince**, **Tom Vendrig**, **Vivian A. den Hertog** and **Michèle N. J. Keizer**: Software. **Tom Vendrig**, **Michèle N. J. Keizer** and **Reinoud W. Brouwer**: Supervision. **Tom Vendrig**: Visualization. **Senja Prince:** Writing—original draft. **All authors**: Writing—review and editing.

## FUNDING INFORMATION

The authors have no funding to report.

## CONFLICT OF INTEREST STATEMENT

The authors declare no conflict of interest.

## ETHICS STATEMENT

The study design, procedure, and protocol received approval from the local Central Ethics Committee (CTc number: 202200331) of the University Medical Center Groningen. Informed consent was obtained from all individual participants included in the study.

## Supporting information

Supporting File 1.

## Data Availability

Data are available upon request.
