# Supplementary material for: Muscle fatigue affects dynamic tibiofemoral movements during jumping tasks in healthy participants
Source: J Exp Orthop. 2026 May 29;13(2):e70794. doi: 10.1002/jeo2.70794 (PMC13240568; doi:10.1002/jeo2.70794)
Supplement: Supplementary file 1 — Supporting File 1. [file JEO2-13-e70794-s001.docx]

**APPENDIX A.** Quantification of kinematic outcomes


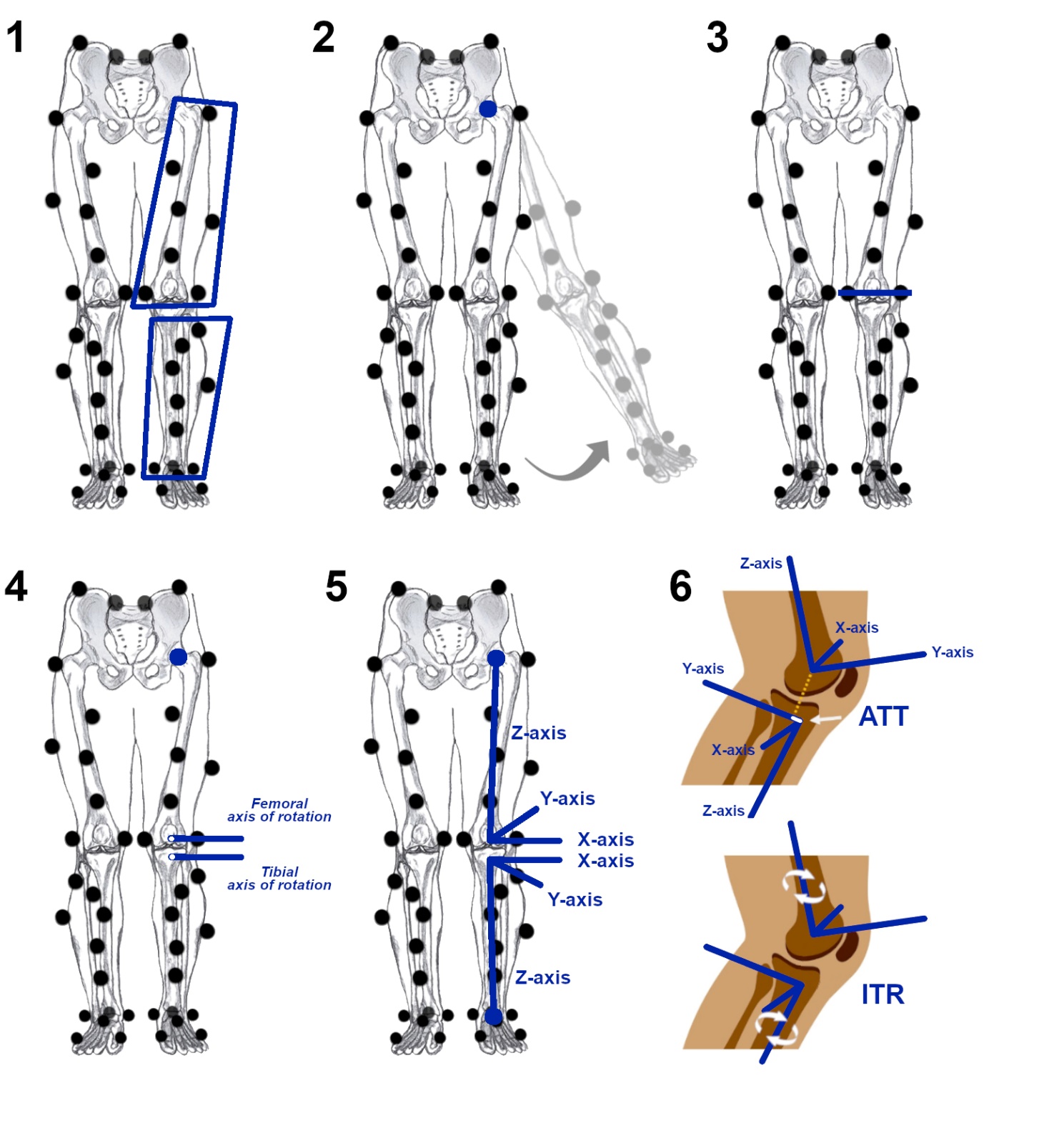


Figure A1: a marker setup was used that is least affected by soft tissue artifacts [4]. Markers were positioned on the posterior superior iliac spine, the iliac crest, the greater trochanter, the medial and lateral epicondyles of the femur, the medial and lateral malleoli of the ankle, the heel, anterior of the talus bone and the first and fifth metatarsophalangeal joints. In addition, four extra markers were placed on the thigh, and six on the shank. A static calibration pose was performed to establish a rigid body marker configuration for the femur and tibia segments separately to further minimize soft tissue artifact, using the optimal common shape technique (OCST) [1, 6].

Figure A2: participants performed a star-arc movement while standing upright to estimate the hip joint center, using the symmetrical center of rotation estimation (SCoRE) [3]. The calculated hip joint center is illustrated as a blue dot.

Figure A3: participants performed a knee flexion-extension movement to estimate the functional flexion-extension axis of rotation, using the symmetrical axis of rotation approach (SARA) [2]. The axis of rotation is illustrated as a blue line.

Figure A4: The calculated functional flexion-extension axis of rotation was duplicated and rigidly linked to two rigid bodies: once to the rigid body of the femur (defined as the femoral axis of rotation) and once to the rigid body of the tibia (defined as the tibial axis of rotation), resulting in two axes whose motion were aligned with the motion of their respective segment. The midpoint of the medial and lateral epicondyle markers was calculated and projected onto the femoral flexion-extension axis of rotation and the tibial flexion-extension axis of rotation, which were defined as the femoral and tibial knee centers, respectively. The remaining vectors (shown in the figure) originate from their respective knee center (white dots on the axes of rotation) and are directed laterally along its respective axis of rotation. For visualization purposes, they are replaced on their corresponding segments with greater seperation from each other, rather than at their true spatial locations.

Figure A5: From these vectors, two separate local, right-handed coordinate systems were constructed: one for the femur and one for the tibia, where the motion of each coordinate system aligns with the motion of its corresponding segment. For the femoral coordinate system, the origin was located at the femoral knee center, with its x-axis defined by the normalized femoral axis of rotation. A temporary z-axis was created by the normalized vector from the femoral knee center to the hip joint center. For the tibial coordinate system, the origin was located at the tibial knee center, with its x-axis defined by the normalized tibial axis of rotation. A temporary z-axis was created by the normalized vector from the tibial knee center to the ankle joint center (defined as the midpoint of the two malleoli markers). For both coordinate systems, the y-axes were generated by computing the cross product of their respective x-axes and temporary z-axes. Finally, orthogonality was ensured by reconstructing the z-axis through the cross product of their respective x-axes and y-axes.

Figure A6: The distance of the origin of the femoral coordinate system relative to the tibial coordinate system was computed in the tibial fixed coordinate system, with dynamic anterior tibial translation (ATTd) measured as the translation along the y-axis of the tibia, as illustrated in the figure. For dynamic internal tibial rotation (ITRd), the rotation matrix from the femoral system to the tibial system was computed by dividing the transpose of the tibial system by the transpose of the femoral system. The Euler rotation angles of the femoral coordinate system relative to the tibial coordinate system were computed to determine knee flexion angle, knee valgus angle and ITRd (Euler sequence: X-Y-Z; flexion/extension - valgus/varus - internal/external rotation) [5]. This method has demonstrated adequate reliability for research purposes [7].

**REFERENCES**

1. Ehrig RM, Heller MO, Kratzenstein S, Duda GN, Trepczynski A, Taylor WR (2011) The SCoRE residual: a quality index to assess the accuracy of joint estimations. J Biomech 44:1400-1404

2. Ehrig RM, Taylor WR, Duda GN, Heller MO (2007) A survey of formal methods for determining functional joint axes. J Biomech 40:2150-2157

3. Ehrig RM, Taylor WR, Duda GN, Heller MO (2006) A survey of formal methods for determining the centre of rotation of ball joints. J Biomech 39:2798-2809

4. Keizer MNJ, Otten E (2020) Technical note: sensitivity analysis of the SCoRE and SARA methods for determining rotational axes during tibiofemoral movements using optical motion capture. J Exp Orthop 7:6

5. Robertson DGE. *Research Methods in Biomechanics.* Human Kinetics; 2004.

6. Söderkvist I, Wedin PA (1993) Determining the movements of the skeleton using well-configured markers. J Biomech 26:1473-1477

7. Vendrig T, Keizer MNJ, Houdijk H (2026) Inter-examiner and inter-day reliability of dynamic tibiofemoral movements measurement using motion capture during walking and jumping tasks. Knee 59:104321
